# “Some are healthy and others not”: Characterization of vended food products by Accra-based food retailers

**DOI:** 10.3389/fpubh.2022.941919

**Published:** 2022-11-03

**Authors:** Silver Nanema, Akosua Adjei, Gideon Senyo Amevinya, Amos Laar

**Affiliations:** Department of Population, Family and Reproductive Health, School of Public Health, University of Ghana, Accra, Ghana

**Keywords:** food acquisition, food environment, food retailers, Ghana, overweight/obesity

## Abstract

**Background and objectives:**

Increasing the availability of healthy foods within food retail outlets can improve consumers' food environments. Such actions or inactions by food retailers may affect people's food purchasing and consumption behavior. This study explored Accra-based food retailers' perceptions and appreciation of “healthiness of food” as a concept. It also documented measures that food retailers adopt to encourage healthy food choices.

**Methods:**

In-person semi-structured interviews were conducted with owners and managers of Accra-based supermarkets (*n* = 7) and corner stores (*n* = 13) in March 2021. The interviews were recorded, transcribed, coded, and analyzed thematically.

**Results:**

The retailers' understanding of healthy food, or lack thereof, is exemplified by such expressions as “health, absence of disease, longevity, balanced diet, diversity, sanitation, and certification.” A handful of retailers described what they sell as “products that meet consumer needs,” “harmless,” or “generally good.” Very few retailers described the food they sell as “junk,” high in sugar, fat, and salt, or energy-dense but nutrient poor foods, or as food that could pose some health risk to consumers. However, some retailers indicated that they advise their customers against the overconsumption of some foods.

**Conclusion:**

Overall, Accra-based retailers have a fair understanding of what constitutes healthy food – exhibiting limited knowledge of the connection between very salty, very sugary, and very fatty foods and health outcomes. Retailers in Accra require interventions that improve their food, health, and nutrition literacy. Improving retailers' food and nutrition literacy may improve the availability of healthier options in food retail outlets in Accra.

## Introduction

The world, and particularly resource-constrained settings, are experiencing multiple forms of malnutrition (characterized by micronutrient deficiencies, childhood undernutrition, and an unprecedented surge in overweight and obesity). In 2016, 70% of the global burden of overweight/obesity was found in Low Middle-Income Countries (LMICs) (1). Agyemang et al. (2) reported an increase in the prevalence of overweight individuals increased by 70% in West Africa between 1990 and 2015. In 2014, the Ghana Statistical Service estimated that over 40% of Ghanaian adults were either overweight or obese (3) with the prevalence of overweight and obesity among Ghanaian children between 9 and 15 years being equally high at 17% (4).

Poor diets are the leading cause of overweight/obesity (5). Itself a non-communicable disease (NCD), obesity is a risk factor for other NCDs such as coronary heart diseases, hypertension, stroke, and diabetes (6, 7). Nutrition transition, characterized by a shift in people's diets from less processed toward highly processed energy-dense foods coupled with reduced physical activity (8), is acknowledged as a contributory factor (2, 9–13).

Poor diets, or nutrition transition, are influenced by food environments. Defined as the collective physical, economic, political, and sociocultural surroundings, opportunities, and conditions that influence people's food and beverage choices and nutritional status (14), food environments include food availability and physical access (proximity); economic access (affordability); promotion, advertising, and information; and food quality and safety. The food environment can be an important entry point for interventions aimed at improving people's diet (15–18). One of the key dimensions of the food environment is the retail food sector – grouped under the typology of built food environment (15). A retail food establishment can be informal or formal depending on whether they are regulated through formal governance structures (19). Typical examples of informal food retail include wet markets, street vendors, kiosks, mobile vendors, and some corner stores. The formal market environments include those regulated through formal governance and typically include supermarkets and hypermarkets (15, 16). Both formal and informal establishments provide the majority of foods that consumers are able to access within the built food environment (20).

Evidence suggests that most supermarkets in Ghana offer highly processed food such as corn flakes, biscuits, cake, and cookies (21); packaged foods such as pasta, sugar, edible oil, coffee, tea, and cocoa drinks; beverages such as sodas, beer, wine, and evaporated and condensed milk; condiments; confectioneries; and minimally processed foods such as maize, flour, or whole wheat bread (21–23). The few supermarkets in Ghanaian cities that offer fresh animal and plant-based food often allocate little shelf or storage space to less healthy food options (21, 24). Other types of retail outlets, such as traditional markets and corner stores, also offer both ultra-processed and fresh food products (23, 24).

The food retail space is expanding rapidly in Ghana, particularly in urban settings (22). However, informal shops and small mobile retailers dominate the retail sector in Ghana. Kroll et al. (23), upon mapping out retail outlets in Ghana's second city Kumasi, reported that formal retailers (i.e., supermarkets) constituted only 7% of retail outlets, while informal shops, stalls, and mobile traders each constituted about 24% of the sample.

Policy actions to improve consumers' food environment by reducing unhealthy diets and increasing the availability of healthy foods within food retail outlets are ongoing in Ghana. These policy actions were considered by the Ministry of Health during a consultative meeting on 30^th^ September 2021 with academics and other experts (25). Knowing the role that food retailers play in influencing people's food purchasing behavior and consumption, it is important that such policy actions are evidence-informed. This study explored Accra-based food retailers' perceptions and understanding of healthy food as well as the measures they adopt to encourage healthy food choices.

## Materials and methods

### Design

This study was cross-sectional and was part of the Measurement, Evaluation, Accountability, and Leadership Support (MEALS4NCDs) project which assessed and supported public sector actions that create healthy food marketing, retail, and provisioning environments in Ghana. Details about the MEALS4NCDs project design and methods are published elsewhere (26).

### Study setting

Interviews were conducted with managers or owners of food retail outlets in the Accra Metropolitan Assembly (AMA) of the Greater Accra Region of Ghana. The Greater Accra Region is the most populous and urbanized region of Ghana and hosts the political capital city Accra. Although the AMA is one of the 29 Metropolitan, Municipal, and District Assemblies (MMDAs) in the Greater Accra Region (27), it is the most urbanized, most populous, and one of the most active sub-administrative districts and business centers. The AMA is home to over 1,800,000 people (28) and receives an influx of over 2,000,000 people daily for socio-economic reasons (27).

### Study population, sampling, and consent procedures

Key Informants (owners or managers) were identified from a list of supermarkets provided by the district (AMA) administrator. The provided list was supplemented with a list of retail shops obtained from Google Maps. The Google search was restricted to three sub-districts in the AMA (Ashiedu Keteke, Okaikoi South, and Ablekuma South) that were purposively selected for the study. The three sub-districts were selected on the basis of level of urbanity population density and level of economic/retail activity relative to other sub-districts. A previous study implemented in the AMA identified these sub-districts as some of the most food retail-dense neighborhoods, with up to 76 street food retailers per sqkm (29).

The initial Google search identified 388 retail outlets. The initial screening and collation eliminated 216 duplicates: 102 without contact details and 41 not meeting our supermarket or comer-store definitions. For this study, a supermarket was defined as a formal food retailer, measuring between 100 and 200m^2^ floor space, with at least one modern cash till, clear aisles, and permitted self-service, while a corner- store was defined as a potentially informal food retailer, measuring 1–5 m^2^ floor space, and only offering over the counter type of service [adapted from (30)]. The distinction between a formal and an informal food establishment was made following the descriptions from Turner et al. (16) and Downs et al. (15). Nine retailers/retail outlets refused to participate in this study. Eventually, 20 Key Informants from seven supermarkets and 13 corner-stores managers/owners were interviewed.

To be interviewed, participants had to be at least 18 years of age and either have ownership of the shop or occupy a managerial position or any position with store-level decisions making capabilities. Informed consent was obtained prior to all interviews.

### Data collection

The interviews were conducted in English either in-person at the retail sites or on the phone depending on the Key Informant's schedule. The interviews were facilitated by two trained field researchers (SN and AA) with prior experience in conducting Key Informant Interviews (KIIs). Interviews lasted between 15 and 45 min.

The Interview guide was divided into 3 sections. **Section A: Knowledge -** consisted of questions relating to what retailers considered healthy food and how they think healthy food contributes to consumers' health. **Section B: Perception -** contained questions on how retailers considered the food they provide in their shop in terms of healthiness. **Section C: Measures -** focused on approaches –if any- retailers have adopted to nudge healthy food choices. The measures recorded were those that apply directly to consumers in the specific retail shop where the interview was conducted.

### Analysis

Each KII was recorded and transcribed verbatim. The transcripts were analyzed using a multistage thematic analysis method (31). Two trained research assistants separately read each transcript to identify themes (see **Tables 2**–**4** for all coding themes and examples). The themes were coded inductively. Coding discrepancies were resolved with a third coder, co-Author AL. All final coding was completed and analyzed in Excel. The coders analyzed each code to identify similarities and differences and to group each code under specific contexts.

## Results

### Characteristics of retail outlets and key informants

Table 1 presents the sociodemographic characteristics of the study respondents. Females represented 60% of the 20 key informants, most of whom are Ghanaians. Corner stores represented 65% of all the retail outlets visited, compared to 35% which were supermarkets. Four of the key informants were owners of their food outlets (*n* = 3 for non-Ghanaian owners). All but one of the corner stores were owned by Ghanaians.

**Table 1:** The sociodemographic characteristic of the respondents.

**Variables**	**Frequency (*n* = 20)**	**Percentage**
**Sex**		
Male	8	40%
Female	12	60%
**Nationality**		
Ghanaian	17	85%
Foreigners (Indian and Lebanese)	3	15%
**Position**		
Manager of a supermarket	3	15%
Manager of a corner store	1	5%
Owner of a supermarket	4	20%
Owner of a corner store	12	60%
**Type of establishment**		
Corner store	13	65%
Supermarket	7	35%

### What do retailers in Accra consider to be “Healthy food”?

In Table 2, we present what Accra-based retailers consider to be healthy food. They associate healthiness with origin or locality; products of Ghanaian origin or those associated with Ghanaian traditions or cultures were rated highly.

“*There's this local food in Ghana called Abom, made with plantain, yam and the stew is made with Kontomire leaves (Cocoyam leaves). This is a typical example [of a healthy food]”*.[**Ghanaian male owner of a supermarket**]

**Table 2:** A landscape of retailers' understanding of healthy food, in Accra.

**Context**	**Theme**	**Example of evidence**
Food Accessibility	Locally available food	“*There's this local food in Ghana called Abom, made with plantain, yam and the stew is made with Kontomire leaves (Cocoyam leaves). This is a typical example [of a healthy food]”*.[**Ghanaian male owner of a supermarket**]
Health	Activity/vitality	“*When you have all the nutrients in you and everything is balanced and you are [able to] exercise well. I mean you will be fine.”* **[Male, Ghanaian manager of a supermarket]**
	Not damaging to health	“*Healthy food is any food that when eaten gives no health problems – now or in the near future”*. [**Ghanaian male owner of a supermarket]**.
	Contributing to long life	“*If you eat healthy food you live long, you grow old. It is very good for you to be on a healthy diet. That is the keto diet.”* [**Ghanaian male manager of a supermarket**]
	Positive impact on health	“*You get good blood circulation, you feel healthy, you feel good.”* [**Ghanaian female owner of a corner store**].
	Protecting health	“*When we eat healthy food, it prevents diseases. Sometimes when you go for [a medical] check-up you may discover that you have excess cholesterol, or obesity. But when you eat healthy food, it limits these things [diseases]. It doesn't eliminate them totally, but it limits them. It [healthy food] gives you a healthy body. Recently I had a problem and when I went for a medical check-up, they [the doctors] said it was cholesterol. The advice I was given was to check on my diet and the way I eat. So, I began to check my diet and I realized that indeed I wasn't eating well”*. [**Ghanaian male manager of a corner store**]
	Therapeutic	“*For example! Diabetes is when your sugar level is up which means you are not eating properly. But, if you eat properly, your cholesterol level will be normal, your diabetes and your sugar level will be normal, and your blood pressure will also be normal.”* [**Ghanaian male owner of a supermarket**]
Nutrition	Balanced diet	“*Healthy food is food that contains all the necessary nutrients that the body needs proportionally”*.[**Ghanaian female owner of a corner store**].
	Diet diversity	“*If you have rice, then you add “kontomire” stew, a little bit of fish like “tuna.” I think you are good to go.”* [**Ghanaian female owner of a corner store**].
	Limited carbohydrate intake	“*We are not supposed to eat too much because too much eating will lead to obesity and all those things [diseases]. Also, too much sugar will cause diabetes. That is what I know”* [**Ghanaian female owner of a corner store**]
	Controlled consumption	“*Food that is high in fat and sugar doesn't really have all the nutrients in them. You don't get anything from them. I can cite pizza for instance. It is more of carbohydrates and just meat.”* [**Ghanaian female owner of a corner store**]
Safety	Food certified by local authorities	“*It [the food] must be approved by [the] Ghana Health Service and I know that [the] Ghana Health Service has developed some standards that they use to teach those [retailers] responsible for handling food stuff and other food items for sale to the public.”* [**Ghanaian Male owner of a corner store**]
	Food hygienically handled	“*If the food is not healthy, those who consume it are bound to fall sick. For example, [from] cholera and other infectious diseases. If the food is not properly handled, it [the disease] may spread through poor handling of food. If those who sell food are infected, they may transmit such diseases to other people. So, it is appropriate that food is properly handled so it wouldn't affect the health of consumers.”* [**Ghanaian male owner of a corner store**]
	Organic food	“*According to me, food like organic food is fresh food.”* [**Indian male owner of a supermarket]**
	Sanatory environment	“*I expect the place to be kept neat. The environment should be neat and clean. That is what I consider.”* [**Ghanaian female owner of a corner store**]
	Food free from contaminants	“*The food must be free of contamination and should be wholesome. If the food has expired, it shouldn't be sold to consumers. Food must be maintained under appropriate temperature to prevent spoilage”* [**Ghanaian male owner of corner store**]

Some retailers indicated that food is considered healthy if it brings some positive health benefits to consumers e.g. “*You get good blood circulation, you feel healthy, you feel good.”* [**Ghanaian female owner of a corner store**]. It was unanimous among retailers that healthy food must not pose any harm to consumers e.g., “*Healthy food is any food that when eaten gives no health problems – now or in the near future.”* [**Ghanaian male owner of a supermarket]**. Retailers in Accra also believe healthy food should have some protective attributes:

“*When we eat healthy food, it prevents diseases. Sometimes when you go for [a medical] check-up you may discover that you have excess cholesterol, or obesity. But when you eat healthy food, it limits these things [diseases]. It doesn't eliminate them totally, but it limits them. It [healthy food] gives you a healthy body. Recently I had a problem and when I went for medical check-up, they [the doctors] said it was cholesterol. The advice I was given was to check on my diet and the way I eat. So, I began to check my diet and I realized that indeed I wasn't eating well”*. [**Ghanaian male manager of a corner store**]

The retailers equate healthy food to a balanced diet. Balanced diet and diet diversity were used interchangeably by retailers. “*Healthy food is food that contains all the necessary nutrients that the body needs proportionally. If you have rice, then you add “kontomire” stew, a little bit of fish like “tuna”. I think you are good to go.”* [**Ghanaian female owner of a corner store**].

Retailers also indicated that healthy food should provide a limited amount of carbohydrates:

*We are not supposed to eat too much because too much eating will lead to obesity and all those things [diseases]. Also, too much sugar will cause diabetes. That is what I know'* [**Ghanaian female owner of a corner store**]

Retailers in Accra associated healthy food with safety as they mentioned terms to reference to certification. A key informant noted:

“*It [the food] must be approved by the Ghana Health Service and I know that the Ghana Health Service has developed some standards that they use to teach those [retailers] responsible for handling food stuff and other food items for sale to the public.”* [**Ghanaian Male owner of a corner store**]

Their understanding of healthy food was also described in relation to hygiene.

“*If the food is not healthy, those who consume it are bound to fall sick. For example, [from] cholera and other infectious diseases. If the food is not properly handled, it [the disease] may spread through poor handling of food. If those who sell food are infected, they may transmit such diseases to other people. So, it is appropriate that food is properly handled so it wouldn't affect the health of consumers.”* [**Ghanaian male owner of a corner store**]

### How do retailers in Accra describe the food they sell with respect to healthiness?

Table 3 presents the opinions of and depictions by Accra-based retailers of the various foods that they provide in their shops. Retailers in Accra describe what they provide as healthy because they are locally obtained.

“*Most of them [the food on sale] are healthy because they are locally made. We know those who make them. We know the type of stuff we provide to consumers”* [**Ghanaian male owner of a supermarket**].

**Table 3:** How retails in Accra describe the food they sell with regard to healthiness.

**Context**	**Theme**	**Example of evidence**
Food Accessibility	Locally available food	“*Most of them [the food on sale] are healthy because they are locally made. We know those who make them. We know the type of stuff we provide to consumers”* [**Ghanaian male owner of a supermarket**].
	Attractively displayed	“*I consider them healthy because of the way they are arranged. Over here [in her shop], you can see the arrangement and the cleanliness [of the food]. The food is either in a tin or in a bottle.”* [**Ghanaian female owner of a corner store**]
Health	Not damaging to health	“*There is no side effect compared to the normal things [food]. There are no side effects.”* [**Ghanaian male manager of a supermarket**]
	Positive impact on health	“*We offer mixed spices with cinnamon and you know that cinnamon is very good for the system [the body]. This is not the regular spices. This is a special one.”* [**Ghanaian Male manager of a supermarket**].
	Therapeutic	“*There is this tea, locally made tea, on one of the shelves that is recommended for people who want to lose weight. They people come back with positive feedback. So, I honestly think that it is working. If it was not working, nobody would come back. They the consumers say. The thing is working fine, my weight is reducing, my appetite is better now”*. [**Ghanaian male owner of a supermarket**]
Nutrition	Balanced diet	“*… they supply the body with proteins, calcium, magnesium, vitamins, all of them.”* [**Lebanese female owner of a supermarket**]
	Both healthy and unhealthy	“*What I can say, some are healthy and others not.”* [**Ghanaian female owner of a corner store**]
	Energy-dense/Junk food	“*Our generation is moving towards junk food. Now we can't do anything because people have changed their habits. They drink coke and all these things a lot. But we are trying to sell diet coke which is good for health. It is better compared to normal coke.” [***Indian male owner of a supermarket]**
		“*In my shop, we offer more of the …[unhealthy] ones. You can't describe them as healthy food because what we sell here are more of snacks and it is just to support you during the course of the day. That shouldn't be your main meal. What we offer here is more of sugar and glucose, which gives energy. But, putting rice and oil together [for example] can give you a balanced diet. So, If you combine food together to have a balanced diet, then you are getting a healthy meal. But if you only rely on food [snacks] such as juice and biscuits, no!”* [**Ghanaian female owner of a corner store**]
	Low in carbohydrate	“*We offer diet coke which has no sugar. Our Tampico for instance is sugar free. So, you can choose this and other natural juices.”* [**Ghanaian Female owner of a corner store]**.
	Plant-based	“*I sell oat, cereal, apple, grapes and we have some mixture of almond and sunflower seed. They are good. When I eat those, I feel good. Sometimes I can't even [stop], I think it's good. But they are expensive and you can't continue eating because of money matter. The cereal costs 50 Ghana cedis (about 4 United States Dollars) which you could use to buy fufu* [a local energy-dense nutrient rich meal]*.”* [**Ghanaian female owner of a corner store**]
	Controlled consumption	“*Drinking too much sugary products can be harmful. Taking too much energy drink can also affect your health. Taking too much alcohol too can also affect your health.”* [**Ghanaian female owner of a corner store**]
	Healthy when combined with other food	“*Yes! [they are healthy]. Oh! I sell rice [raw rice] and rice is a healthy food when you prepare it and add some salad. It is healthy food.”* [**Ghanaian female owner of a corner store**]
Safety	Approved by the retailers themselves	“*I eat some. So, I assumed it is good. But I can't tell where if you eat this one it will be good for you.”* [**Ghanaian female owner of a corner store**]
	Certified by local authorities	“*Well, they are healthy because I believe there are representatives in this country who were given the assignment to approve things [food] that are healthy for supermarkets to be able to sell. The supermarket has a policy that if you are not registered with the FDA you can't come to us for any business. Based on that, I think what we are selling is healthy.”* [**Ghanaian female owner of a corner store**]
	Has caused no complaint yet	“*I will be worried if I get complaints. But no complaint so far for so many years. They [consumers] have come and bought things here, but no one has come and complained. And since it is a supermarket, it is being checked. It is not like a regular store that [is not checked].”* [**Ghanaian male manager of a supermarket**]
	Organic/natural	“*They are organic and healthy for the system.”* [**Ghanaian Male manager of a supermarket**]
	Risky to health	“*The effects are not immediate. Maybe [in] your 60s then the food will give you sugar [diabetes], blood pressure and so other many things. That is what the doctor told me. He asked me why do I like Lucozade? [an energy drink]. He asked me to stop and now I have stopped. Once somebody [the doctor] told me that it is not good, it is [not] good.”* [**Ghanaian female owner of a corner store**]
	Sold in sanitary conditions	“*I think it is ok! They are kept in a neat [environment], and those who bring the bread put them in plastic bags. So, they are protected.”* [**Ghanaian male owner of corner store**]
	Sold as wholesome	“*They are healthy because, as a supermarket, we check where they come from. We check the expiring date of the products we sell here. When they are about to expire we know it's no longer good for consumption, so, we remove them from the shelf. We alert the supplier and they come for it. So, we don't sell things that are not healthy here.”* [**Ghanaian female owner of a corner store**]
Unclear responses	Fitting consumers' needs	“*You can't tell anybody what he should be eating. You can't blame anybody for their choices. We do prefer them to buy more healthy food like fruit juices which are good for health. But it is up to their choice.”* [**Indian male owner of a supermarket**]
	Generally good	“*Topnotch” local, and quality. Three [terms].”* [**Ghanaian male owner of a supermarket**]
		“*It is very healthy ………..”* [**Ghanaian Male manager of a supermarket**]
		“*They are healthy!”* [**Ghanaian female owner of a corner store**]

Other retailers consider what they sell healthy because of the deliberate efforts they invest in displaying the foods attractively. “*I consider them healthy because of the way they are arranged. Over here [in her shop], you can see the arrangement and the cleanliness [of the food]. The food is either in a tin or in a bottle.”* [**Ghanaian female owner of a corner store**]

Some of the retailers claimed that the food they provide is therapeutic:

“*There is this tea, locally made tea, on one of the shelves that is recommended for people who want to lose weight. They people come back with positive feedback. So, I honestly think that it is working. If it was not working, nobody would come back. They the consumers say, “The thing is working fine, my weight is reducing, my appetite is better now””*. [**Ghanaian male owner of a supermarket**]

Some retailers claimed that what they sell contributes positively to health: “*We offer mixed spices with cinnamon and you know that cinnamon is very good for the system [the body]. This is not the regular spices. This is a special one.”* [**Ghanaian Male manager of a supermarket**]. Other retailers described what they sell as plant-based and healthy, but have presented the cost of such food as unaffordable and inaccessible to consumers.

“*I sell oats, cereal, apple, grapes, and we have some mixture of almond and sunflower seeds. They are good. When I eat those, I feel good. Sometimes I can't even [stop], I think it's good. But they are expensive and you can't continue eating because of money matters. The cereal costs 50 Ghana cedis (about 4 United States Dollars) which you could use to buy fufu* [a local energy-dense nutrient rich meal]*.”* [**Ghanaian female owner of a corner store**]

Further, retailers described what they sell as low in added sugar: “*We offer diet coke which has no sugar. Our Tampico for instance is sugar free. So, you can choose this and other natural juices.”* [**Ghanaian Female owner of a corner store]**.

Other retailers consider the food they sell healthy because they have not yet received complaints about that food from consumers.

“*I will be worried if I get complaints. But no complaint so far for so many years. They [consumers] have come and bought things here, but no one has come and complained. And since it is a supermarket, it is being checked. It is not like a regular store that is not checked].”* [**Ghanaian male manager of a supermarket**]

A few retailers indicated that their foods may have negative outcomes on consumers' health and described what they sell as junk food:

“*Our generation is moving towards junk food. Now we can't do anything because people have changed their habits. They drink coke and all these things a lot. But we are trying to sell diet coke which is good for health. It is better compared to normal coke.” [***Indian male owner of a supermarket]**

Some retailers described what they sell as having potential health risks to consumers.

“*The effects are not immediate. Maybe [in] your 60s then the food will give you sugar [diabetes], blood pressure, and so [many other] things. That is what the doctor told me. He asked me why do I like Lucozade? [an energy drink]. He asked me to stop and now I have stopped. Once somebody [the doctor] told me that it is not good, it is [not] good.”* [**Ghanaian female owner of a corner store**]

Another retailer described the food she sells as unhealthy but when combined with other food can be considered healthy.

“*In my shop, we offer more of the …[unhealthy] food. You can't describe them as healthy food because what we sell here are more of snacks and it is just to support you during the course of the day. That shouldn't be your main meal. What we offer here is more of sugar and glucose, which gives energy. But, putting rice and oil together [for example] can give you a balanced diet. So, if you combine food together to have a balanced diet, then you are getting a healthy meal. But if you only rely on food [snacks] such as juice and biscuits, no!”* [**Ghanaian female owner of a corner store**]

Some retailers advised against overconsuming the foods they sell: “*Drinking too much sugary products can be harmful. Taking too much energy drink can also affect your health. Taking too much alcohol too can also affect your health.”* [**Ghanaian female owner of a corner store**]

### What measures have retailers in Accra put in place to nudge healthy food choices?

Finally, Table 4 presents the measures retailers indicated implementing at the shop level to encourage healthy food purchases. Most retailers in Accra provide oral advice to nudge consumers towards adopting healthy food choices.:

“*Normally when they come, somebody will come and tell you, maybe “I want a sugar-free drink” and I tell them maybe you can get “cerees” because it is sugar-free. But I think “purejoy” at times they add a little sugar, not 100% sugar.”* [**Ghanaian female owner of a corner store**]

Some retailers indicated providing advice to consumers only upon request by consumers. “*Some people would say they don't want a drink that contains lots of sugar. So, if you have the one [in which] the sugar is less, you recommend it to them. They normally ask.”* [**Ghanaian Female owner of a corner store**]

Some retailers indicated refusing to sell some food items to some consumers when they realize that those consumers overconsume the food they sell. e.g. “*When I see that you are taking it too much, I don't sell it to you again. That is what I do. When they come, I don't sell it? That is what I do.”* [**Ghanaian female owner of a corner store**].

**Table 4:** What measures retailers in Accra have in place to nudge healthy food choices.

**Context**	**Theme**	**Evidence of example**
Retailers take no action	Costumers exercise caution when shopping	“*You know in “Tesanon” [neighborhood] here, our consumers are educated. So, sometimes they can google the product and find [out] if the product is registered. So, we don't have that much challenges because most of the consumers are very educated. At times if it a product is getting close to its expiry date they [the costomers] mostly inform us. So, at times they draw our attention. So we don't find that difficulty with that.”* [**Ghanaian male manager of a supermarket**]
Proactive retailers acting driven by their own initiative	Outsource for local food	“*We go to the market every day and try to find locally made stuff that we know their origin or source. For example, we know that this is made of “sesame seed” product and it's from China. We try to go to the local market to find out if we can get “sesame seed” product that is locally made before we know the exact process it went through before coming to us. We are making endless efforts, my team and I.”* [**Ghanaian male owner of a supermarket**]
	Provide health related advice	“*Sometimes when I warn them, they say “this one has no sugar added” and when they talk like that, some of them say “my doctor said I should take it” because coke some has no-sugar.”* [**Ghanaian female owner of a corner store**]
	Attractively display the food products	“Displaying plays a big role. As you see the dates for instance, we are selling plenty dates every day because we are displaying them in a very attractive way. It can attract the customer even if he doesn't need it, he will buy it. So, displaying has a big role. Also, if the customer as I told you, needs our advice we will give him the real advice” [**Lebanese female owner of a supermarket**
		“*This shelf is healthy. This stand is all zero sugar items. They don't contain sugar. The beans also, are all healthy for the body. But the chocolate stands are mostly the sugar stand. But people like them. They select from the stand according to their need and their demand.”* [**Lebanese female owner of a supermarket**]
Retailers Reacting only to consumers' demands	Restrict the purchase of certain food	“*When I see that you are taking it too much, I don't sell it to you again. That is what I do. When they come, I don't sell it? That is what I do.”* [**Ghanaian female owner of a corner store**].
	Provide advice on healthy food options	“*Normally when they come, somebody will come and tell you, maybe “I want a sugar free drink” and I tell them maybe you can get “cerees” because it is sugar-free. But I think “purejoy” at times they add a little sugar, not 100% sugar.”* [**Ghanaian female owner of a corner store**]
		“*Some people would say they don't want a drinks that contain lots of sugar. So, if you have the one that the sugar is less, you recommend it to them. They normally ask.”* [**Ghanaian Female owner of a corner store**]
		“*Quite a few of them ask question [express need for advice]. When they enter they [ask] “can you help me shop?” and as you are going with them you advise them” I heard this is good. How good is it?” you are helping him. Only a few ask.”* [**Ghanaian male manager of corner store**]
		“*We have drinks that do not contain sugar and so, some people when they come, they request for sugar-free things. For example, if we has sugar-free biscuits and sugar-free drinks. So, when they come and request for sugar free foods, we offer them here.” [***Ghanaian female owner of a corner store***]*

Some retailers demonstrated proactiveness as they indicated making efforts to carefully select the food products they believe are healthy for their customers:

“*We go to the market every day and try to find locally made stuff that we know their origin or source. For example, we know that this is made of “sesame seed” product and it's from China. We try to go to the local market to find out if we can get “sesame seed” product that is locally made before we know the exact process it went through before coming to us. We are making endless efforts, my team and I.”* [**Ghanaian male owner of a supermarket**]

Some retailers indicated making attempts to display what they consider healthy food in an attractive way. “Displaying **plays** a big role. As you see the dates, **for instance, we** are selling plenty [of] dates every day because we are displaying **them** in a very attractive way. It can attract the customer **e**ven if he doesn't need it, he will buy it. So, displaying has a big role. Also, if the customer, as I told you, need**s** our advice we will give him the real advice” [**Lebanese female owner of a supermarket**].

## Discussion

This study aimed to elucidate retailers' understanding of healthy food, their perceptions of the healthiness of the food they vend, and the measures that they adopt to encourage healthy food choices. Overall, Accra-based food retailers' characterization of what they sell is similar to the current scientific depictions of healthy food. The retailers described healthy food as edible substances that provide positive health outcomes to consumers and which should not cause or contribute to illness. In their descriptions, they mentioned terms like “balanced diet” and “diversity,” or referred to the nutrient profile that a healthy food should have. Currently, some of the science-based models for classifying food as healthy or unhealthy are nutrient-based (32, 33), food-based (34), or based on the level of processing (35).

To illustrate, in the US, the FDA defines healthy food as based on the nutrition profile of the food. They consider healthy food to be food products low in total fat and saturated fat, and which provide at least 10% of the recommended daily intake of certain vitamins and minerals (36). The International Network for Food and Obesity/Non-communicable Diseases Research, Monitoring and Action Support (INFORMAS) classifies foods as healthy based on their nutrient profile. INFORMAS has developed five food groups considered “core”: grains and grain products; vegetables and legumes/beans; fruits; milk and milk products; lean meat, fish, poultry, eggs, nuts, and legumes (37). Foods considered less healthy are referred to as non-core and included sweet breads, cakes, sweet biscuits, high fat savory biscuits, pastries, tinned meats, sweet snack foods, chips, extruded snacks, ice cream, etc. A third category, miscellaneous, includes foods such as recipe additions e.g., cubes, oils, dried herbs, tea, and coffee (excluding sweetened powder-based teas and coffees), etc., (37).

Retailers in Accra associated healthy food with safety, origin, and accessibility. They mentioned dimensions such as sanitation, hygiene, certification, and country of origin as important. Food safety concerns affect consumer behaviors and diets in low and middle-income countries (38) and those based in Accra as well (13). According to Liguori et al. (38), such concerns include fear of pesticides, hygiene in/around food outlets, unhygienic vendor practices, and household storage/preparation methods. These concerns may increase the stocking, vending, and consumption of less healthy starchy staples and processed/packaged foods compared to fresh fruit and vegetables.

Accra-based food retailers did not consider the extent of processing as a criterion for healthiness. The NOVA System classifies food based on the extent of processing (35). Although this classification system does not directly make any distinction about what food is healthy or not, individuals can use it to make important health-related decisions. Classifying foods from unprocessed/minimally processed foods (relatively healthier) through to ultra-processed foods (relatively less healthy), the NOVA system recommends that consumers limit their intake of ultra-processed foods compared to unprocessed and minimally processed foods.

As to the extent of availability and stocking of ultra-processed foods in food retails outlets of Accra, a related study conducted in Accra provides evidence (21). The study estimated that, for every 1 m^2^ of shelf space allotted to healthy foods, there was 6 m^2^ equivalent of space allocated to less healthy foods or ultra-processed foods. It is possible that these stocking practices observed in the supermarkets or retail shops in Accra may be a response to consumers' demand for less healthy food. Indeed, respondents in the current study mentioned that their food stocking practices correlated with consumer demand.

Some retailers in Accra adopt limited measures to encourage consumers to adopt healthy food choices. Some of their efforts include sourcing foods from local wet or open markets for healthy food. Most retailers indicated providing guidance on healthier food options they have, but only if their customers expressed the need.

Globally, scholars and practitioners are now suggesting encouragement as a promising intervention to increase healthy food consumption (39). The potential of encouragement to increase healthy food decisions was underscored by Arno and Thomas (40) who showed that this leads to an overall increase in healthier food choices by 15.3%. Van Gestel et al. (41) also reported that repositioning healthy food products at the checkout counter increases the sales of those healthy food products. Their findings imply that there are clear opportunities to improve food choices by acting directly on the retail sector (15). Those opportunities can be seized by deploying a mix of high agency interventions that improve nutrition literacy and healthy food choices through nudges or counter-marketing strategies as well as low agency interventions zoning regulations, food-related fiscal policies, and improvement of food retail environments. To clarify, intervention agency usually refers to the resources (e.g., personal, psychological, cognitive, financial, material) on which individuals draw to engage successfully with an intervention (42). Those interventions that require individuals to invest fewer individual personal and psychological resources are described as low agency (fortification of food with micronutrients, marketing restrictions, fiscal policies, and product reformulations). In contrast, high agency interventions such as educational interventions, mass media campaigns, or front of pack labelling require substantial agency in terms of accessing, understanding, and applying the information provided (42).

## Limitations

Our study is not flawless. Participating stores were identified and recruited from a purposive sample rather than through a probabilistic sampling approach. It is also possible that managers/owners who agreed to participate in the study were those with a predilection for healthy food retail. A large proportion of our sample were corner stores and a small fraction consisted of independent supermarkets. No chain supermarkets were included in our study. This limits the generalizability of our findings to other types of supermarkets and food retail outlets. Besides, this study did not cover rural settings/communities in Ghana. Such areas may have unique challenges not captured in our study. Therefore, the findings of this study need to be interpreted or extrapolated with the stated limitations and delimitations in mind.

## Conclusions

The Accra-based retailers included in this study have a fair understanding of what constitutes healthy food. Some of the retailers employ limited measures to encourage healthy food stocking and choices. There is an opportunity for policy and interventions in the food retail sector in Ghana. Such interventions may improve consumers' dietary choices.

## Recommendations

We recommend nutrition literacy interventions targeted at food retailers as well as retail-level training programs that seek to inculcate healthy retail strategies, such as nudging, to retailers. Given that Ghana is in the middle of a food environment policy reform, addressing these gasps in the retail sector will further enhance the anticipated success of the food environment transformation in Ghana.

## Data availability statement

The raw data supporting the conclusions of this article will be made available by the authors, without undue reservation, and upon reasonable request.

## Ethics statement

The studies involving human participants were reviewed and approved by the Ethics Review of the Humanities, University of Ghana (Approval # ECH 152-18-19), and the Ghana Health Service Ethical Review Committee (Approval # GHS-ERC 005-06-19). The patients/participants provided their written informed consent to participate in this study.

## Author contributions

AL conceived and led the design of the study. SN, AA, and GA contributed to the design. AL and GA supervised the implementation of the fieldwork. SN and AA conducted the interviews with retailers and transcribed and coded them. GA supported data management and analysis. SN drafted the manuscript and received significant and relevant inputs from AL. All authors contributed to the article and approved the submitted version.

## Funding

This paper was supported by the Measuring the Healthiness of Ghanaian Children's Food Environments to Prevent Obesity and Non-communicable Diseases (MEALS4NCDs) Project and funded by the International Development Research Centre (IDRC) Food, Environment, and Health Programme–IDRC, Canada (Grant #108983).

## Conflict of interest

The authors declare that the research was conducted in the absence of any commercial or financial relationships that could be construed as a potential conflict of interest.

## Publisher's note

All claims expressed in this article are solely those of the authors and do not necessarily represent those of their affiliated organizations, or those of the publisher, the editors and the reviewers. Any product that may be evaluated in this article, or claim that may be made by its manufacturer, is not guaranteed or endorsed by the publisher.
